# Upregulation of ubiquitin-conjugating enzyme E2T (UBE2T) predicts poor prognosis and promotes hepatocellular carcinoma progression

**DOI:** 10.1080/21655979.2021.1918507

**Published:** 2021-05-03

**Authors:** Xiaoyue Ren, Alex Li, Edward Ying, Jhin Fang, Mingzhu Li, Jiao Yu

**Affiliations:** a Department of Radiotherapy, Shaanxi Provincial People’s Hospital, Xi’an, Shaanxi, China; b Department of Hepatobiliary Surgery, The Affiliated People’s Hospital of Ningbo University, Ningbo, Zhejiang, China

**Keywords:** HCC, UBE2T, miR-212-5p, migration

## Abstract

Reportedly, ubiquitin-conjugating enzyme E2T (UBE2T) is closely related to the progression of several malignancies. This work is aimed to probe the role of UBE2T in the progression of hepatocellular carcinoma (HCC) patients. The microarray analysis was executed to screen the differentially expressed genes (DEGs) in HCC tissues. The Cancer Genome Atlas (TCGA) and Gene Expression Profiling Interactive Analysis (GEPIA2) databases, PCR and immunohistochemistry were utilized to validate the dysregulation of UBE2T in HCC. Kaplan-Meier analysis was employed to determine the relationship between UBE2T expression and the prognosis of HCC patients. PCR was carried out to detect UBE2T protein expression in HCC cell lines. Cell Counting Kit-8 (CCK-8) assay and 5-bromo-2ʹ-deoxyuridine (BrdU) experiments were conducted to examine the proliferation of HCC cells. Scratch healing and Transwell experiments were conducted to examine the migration of HCC cells. Bioinformatics analysis and dual-luciferase reporter gene experiments predicted and validated the targeting relationship with miR-212-5p and UBE2T. We found that UBE2T expression was remarkably up-modulated in HCC tissues and cell lines, and its high expression was linked to a worse prognosis in HCC patients. UBE2T overexpression enhanced HCC cell proliferation and migration. Additionally, UBE2T was verified as a downstream target of miR-212-5p. In conclusion, UBE2T overexpression is markedly linked to unfavorable prognosis in HCC patients. UBE2T, regulated by miR-212-5p, significantly enhances the malignant phenotypes of HCC cells, which can be used as a target for HCC diagnosis and prognosis.

## Graphical abstract

**Figure:**
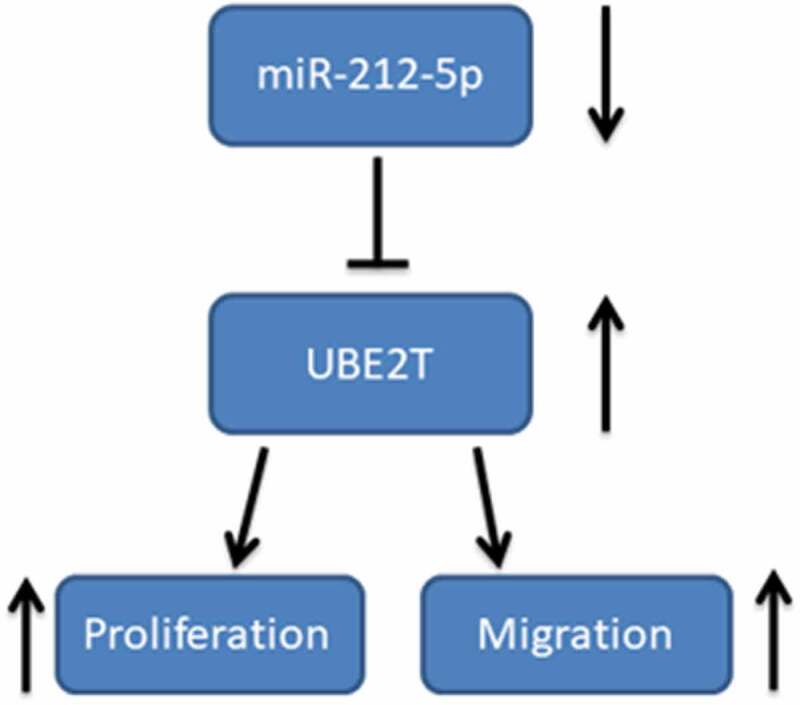

## Introduction

Hepatocellular carcinoma (HCC) is the most common histological subtype of liver cancer, taking up around 90% of primary liver cancer cases [1]. Statistically, HCC has become the third major cause of cancer-related death worldwide [2]. Although liver resection, liver transplantation, chemotherapy, and radiotherapy can prolong the survival of patients with HCC, the high recurrence rates and distant metastasis of HCC still result in low survival and adverse prognosis [3]. Therefore, understanding the molecular regulatory mechanisms in HCC is critical.

Ubiquitin-conjugating enzyme E2T (UBE2T) belongs to the ubiquitin-conjugating (E2) enzyme family that is crucial in the ubiquitin-proteasome pathway [4]. Reportedly, UBE2T is vital in the ubiquitination of proteins, which is essential to modulate many biological processes, including inflammation, immune response, division of cells and multiplication of cells [5]. UBE2T overexpression is reported to lead to unfavorable prognosis in patients with gallbladder cancer and UBE2T can be used as an independent prognostic biomarker for patients with gallbladder cancer [5]. UBE2T overexpression facilitates the growth, proliferation, and invasion of colorectal cancer cells and suppresses apoptosis [6]. Nonetheless, the role of UBE2T in HCC and its regulatory mechanisms need to be further elucidated.

MicroRNAs (miRNAs) are endogenous, small, non-coding RNAs that modulate gene expression by interacting with messenger RNA (mRNA), leading to mRNA degradation or restraining its translation [7]. MiRNAs are implicated in multiple signaling pathways that modulate cell cycle, multiplication, differentiation, and apoptosis, and thus they have been considered as therapeutic targets in tumors [8]. For instance, miR-616 enhances HCC cell proliferation, migration, and invasion by inhibiting phosphatase and tensin homolog (PTEN) expression and activating the phosphatidylinositol-3 kinase (PI3K)/AKT pathway; miR-616 high expression is positively linked to lymph node metastasis and Tumor-Node-Metastasis (TNM) staging [9]. A previous study confirms that UBE2T is directly targeted by miR-543, which is down-regulated in HCC [10]. Another study shows that miR-1305 targets UBE2T to inhibit the AKT pathway, thereby suppressing the self-renewal and tumorigenicity of live cancer stem cells [11]. Additionally, miR-212-5p expression is remarkably reduced in HCC [12]. Nevertheless, little has been reported about the mechanism by which miR-212-5p modulates HCC progression.

Diverse genes are unveiled to be linked to the progression and prognosis of HCC, and the identification of specific differentially expressed genes is therefore essential for clarifying the molecular mechanism of HCC progression [13]. The Cancer Genome Atlas (TCGA) and Gene Expression Omnibus (GEO) are usually employed to probe the molecular basis of heterogeneity and progression of HCC and identify novel biomarkers linked to hepatocarcinogenesis [14].

The present study aims to explore the relationship between UBE2T and the progression of HCC, and to further explore the mechanism of miR-212-5p regulating UBE2T in HCC. We report that miR-212-5p and UBE2T are promising biomarkers and targets for HCC diagnosis and therapy.

## Materials and methods

### Subjects and specimens

25 men and 11 women who were diagnosed with HCC at the Affiliated People’s Hospital of Ningbo University and underwent surgical resection were included in this research. There were 18 patients over 60 years old and 18 patients under 60 years old. Among them, 16 cases were well-differentiated and 20 cases were poorly differentiated. According to TNM staging criteria, 21 patients were at T1 or T2 stage, and 15 were at T3 or T4 stage; 13 patients had lymph node metastasis, whereas 23 did not. HCC tissue and adjacent normal tissue were obtained during the surgery and immediately preserved in liquid nitrogen. This work was endorsed by the Ethics Committee of the Affiliated People’s Hospital of Ningbo University and written informed consent was available from the patients.

### Microarray data

The microarray data were acquired from the GEO database. Three gene expression datasets (GSE64041, GSE74656, and GSE76427) were used. GEO2R data analysis tool was employed to screen the differentially expressed genes (DEGs) between normal liver tissue specimens and HCC specimens. *P* < 0.05 and |log2(Fold Change)|>1 were defined as the thresholds for the screening of the DEGs. Volcano plots displayed the up-modulated and down-modulated DEGs, and Venn diagrams was used to further screen the DEGs which were dysregulated in all of the three datasets.

### Cell culture and transfection

Human HCC cell lines (Hep3B, HepG2, SK-help1, and PLHC-1) and human normal hepatocyte cell line (THLE-3) were procured from the American Type Culture Collection (ATCC, Manassas, VA, USA). All cells were cultured in RPMI-1640 medium (Gibco, Carlsbad, CA, USA) containing 10% fetal bovine serum (FBS) (Gibco, Carlsbad, CA, USA) and 100 U/mL penicillin, and 100 μg/mL streptomycin (Invitrogen, Carlsbad, CA, USA) at 37°C with 5% CO_2_. pcDNA3.1-UBE2T, empty vector, UBE2T siRNA (si-UBE2T#1/si-UBE2T#2), scramble siRNA, miR-212-5p mimics, miR-212-5p inhibitors and negative controls (mimics NC/inhibitors NC) were available from Invitrogen (Carlsbad, CA, USA). Hep3B and HepG2 cells were transfected with Lipofectamine® 2000 (Invitrogen, Carlsbad, CA, USA) according to the manufacturer’s instructions.

### Quantitative Real-time PCR (qRT-PCR) analysis

HCC cells were harvested and total RNA was extracted using TRIzol Reagent (Yeasen Biotech, Shanghai, China). cDNA synthesis was executed using the TaqMan MicroRNA reverse transcription kit (Applied Biosystems, Foster City, CA, USA) for miR-212-5p and employing PrimeScript RT Master Mix Kit (Takara Bio, Tokyo, Japan) for UBE2T, respectively. Then with cDNA as the template, qRT-PCR was executed to determine UBE2T relative expression with the SYBR® Premix Ex TaqTM II (Takara Bio, Tokyo, Japan). Meanwhile, stem-loop primer SYBR Green qRT-PCR (Synbio Tech, Suzhou, China) was applied for qRT-PCR to evaluate miR-212-5p expression. With U6 and β-actin as the internal references, miR-212-5p and UBE2T relative expression were calculated by 2^−ΔΔCt^ method. Primer sequences were listed in Table 1.

**Table 1.** Primer sequences

Name	Primer sequences
miR-212-5p	Forward: 5’-GCTTACGCTTCGAGCCCAC-3’
	Reverse: 5’-GACACCACGGCCCACTCTGCA-3’
UBE2T	Forward: 5’-TTGATTCTGCTGGAAGGATTTG-3’
	Reverse: 5’-CAGTTGCGATGTTGAGGGAT-3’
U6	Forward: 5’-GCTTCGGCAGCACATATACTAAAAT-3’
	Reverse: 5’-CGCTTCAGAATTTGCGTGTCAT-3’
β-actin	Forward: 5’-TGGCACCCAGCACAATGAA-3’
	Reverse: 5’-CTAAGTCATAGTCCGCCTAGAAGCA-3’

### Immunohistochemistry (IHC) staining

Briefly, the tissue sections were dewaxed using xylene, gradient alcohol, and distilled water, then boiled in 10 mM sodium citrate buffer (pH 6.0) at 95–100°C for 30 min. After blocking with 10% goat serum for 1 h, the tissue sections were incubated overnight at 4°C with anti-UBE2T antibody (cat. no. ab154022, Abcam, 1:100), and then incubated with the secondary antibody (cat. no. ab190475, Abcam; 1:500) for 30 min at room temperature. After rinsing again with PBS, the sections were stained with DAB (Beyotime Institute of Biotechnology). The IHC staining results were independently and blindly scored by two pathologists

### Cell counting kit-8 (CCK-8) experiment

Cells (2 × 10^3^ cells/well) were seeded in 96-well plates, each well containing 100 μL of medium, and three parallel wells were set up. 10 μL of CCK-8 solution (Beyotime Institute of Biotechnology, Shanghai, China) was supplemented to each well at the indicated time points (0 h, 24 h, 48 h, 72 h, 96 h). After the cells were incubated at 37°C for 2 h, the absorbance at 450 nm was measured with a microplate reader (Model 550; Bio-Rad Laboratories, Inc., Hercules, CA, USA).

### BrdU proliferation experiment

HCC cells (1 × 10^5^ cells/mL) were planted in 35-mm diameter culture dishes and cultured for 1 day. Next, 1.0 mg/ml of BrdU reagent (BD Pharmingen, San Diego, CA, USA) was supplemented and the cells were incubated for 4 h at 37°C. The medium was discarded, and the slides were rinsed 3 times with PBS and fixed with methanol for 10 min. After drying the slides, the endogenous oxides were inactivated and the nonspecific antigens were blocked with 5% rabbit serum. After DNA denaturation, the cells were rinsed with PBS, and the primary antibody was supplemented, and the cells were incubated for 1 h. Thereafter, the cells were incubated with the secondary antibody for 1 h. Subsequently, the cells were stained with DAPI staining solution (Beyotime Institute of Biotechnology, Shanghai, China). BrdU-positive cells in 3 randomly selected fields of view were counted under the microscope.

### Wound healing experiment

HCC cells (1x10^4^ cells/well) were planted in 6-well plates, and when the confluency of the cells reached to 90%, the tip of the 200 μL sterile pipette was used to make a “one’ scratch in the middle of the plate. The cells were then gently rinsed 3 times with serum-free medium, and then cultured with serum-free medium. Photographs were taken at 0 h and 24 h after the formation of scratches, and the scratch widths were recorded.

### Cell migration experiment

The cells were trypsinized with 0.25% trypsin and made into single cell suspension with serum-free medium, and the cell concentration was modulated to 1 × 10^5^ cells/ml. 200 μL of cell suspension was supplemented to the upper compartment of each Transwell chamber (Corning, Shanghai, China), and 500 μL of medium containing 10% FBS was supplemented to the lower compartment and then the cells were incubated in a 5% CO_2_ at 37°C for 24 h. The cells passing through the filter were fixed with methanol and stained with 0.1% crystal violet. Five fields of view were randomly selected under the microscope, and the migration of tumor cells was indicated by the number of the cells.

### Western blot

The cells were lysed with RIPA lysis buffer (Solarbio, Beijing, China), and the supernatant of the lysate was harvested. The BCA protein assay kit (Beyotime, Haimen, China) was exploited to determine the protein concentration. The proteins were separated by SDS-PAGE and transferred to a PVDF membrane. After blocking the membrane with 5% skim milk for 1 h, the anti-UBE2T antibody (cat. no. ab154022, Abcam, 1:1000) and anti-GAPDH antibody (cat. no. ab8245, Abcam, 1:1000) were added, and the membrane was incubated with the antibodies overnight at 4°C. The next day, the membrane was incubated with horseradish peroxidase-conjugated secondary antibody for 50 min at room temperature. Finally, the protein bands were developed employing an Amersham Imager 600 (GEHealthcare, Chicago, IL, USA) with an electrochemiluminescence kit (Biosharp, Heifei, China).

### Luciferase reporter experiment

Bioinformatics analysis was adopted to predict the binding sequence between miR-212-5p and UBE2T 3’ UTR, and PCR was implemented to amplify the binding fragment between UBE2T and miR-212-5p. The UBE2T 3’UTR wild-type (wt) plasmid was constructed by inserting the amplification products into the pGL3-Promoter plasmid vector, and the UBE2T 3’UTR mutant (mut) plasmid was constructed by site-directed mutagenesis of the binding fragment. HEK-293 T cells were co-transfected with the reporter vectors and miR-212-5p mimics (or mimics NC), respectively. 48 h after the transfection, the cells were harvested. Fluorescence values were determined using a dual-luciferase reporter gene assay system (Promega, Madison, WI, USA) under the guidance of the protocols.

### Statistical analysis

All of the experiments were performed in triplicate. The data were presented as ‘mean ± SD’ and were statistically analyzed using SPSS 20 software (SPSS, Abbott Laboratories, Chicago, IL, USA) and GraphPad Prism 8 (GraphPad Software, Inc., La Jolla, CA, USA). Comparisons between two groups were conducted by independent samples *t*-test, and comparisons of means between multiple groups were executed by one-way ANOVA. Overall survival analysis was executed with Cox multivariate regression analysis and log-rank tests. Pearson correlation coefficient analysis was carried out to analyze the correlation of gene expression levels. *P* < 0.05 signified statistical significance.

## Results

### Identification of DEGs in HCC based on bioinformatics analysis

**Figure 1.**
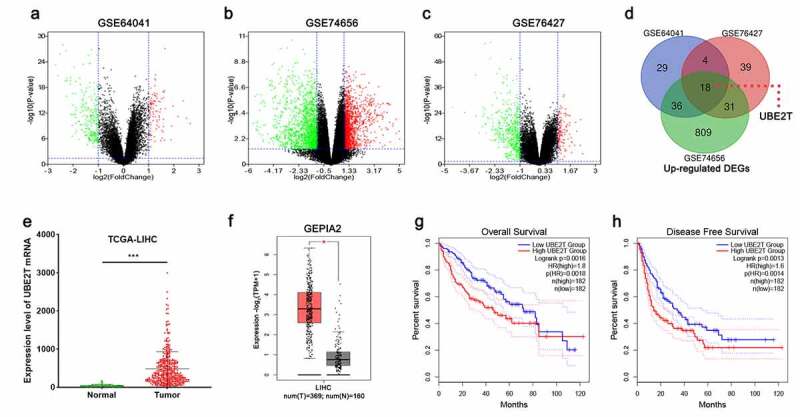
Identification of DEGs based on bioinformatics analysis. (a-c) Volcano plots of DEGs in the GSE64041, GSE74656, and GSE76427 datasets. (d) Venn diagrams illustrated the number of up-modulated DEGs in the three gene expression datasets. (e and f) TCGA and GEPIA2 databases were utilized to analyze UBE2T expression in HCC. (g and h). GEPIA2 database was adopted to analyze the relationship between UBE2T expression and overall survival and disease-free survival of HCC patients

In order to explore the abnormal expression of genes in HCC, three gene expression datasets, GSE64041, GSE74656, and GSE76427, were obtained from the GEO database and analyzed online using GEO2R tool for DEGs between normal liver tissues and HCC tissues. The volcano plots showed the gene expression profiles of the three datasets, with green representing down-modulated genes and red representing up-modulated genes (Figure 1a-Figure 1c). Additionally, Venn diagrams were employed to show the genes with up-modulated expression in all of the three datasets, and 18 genes were obtained, including UBE2T (Figure 1d). Data were then obtained from the TCGA database and the GEPIA2 database for validation, and the data indicated that UBE2T expression in HCC tissues was remarkably higher than in normal tissues (Figure 1e and Figure 1f). Moreover, the data of the GEPIA2 implied that UBE2T overexpression was remarkably linked to lower overall and disease-free survival in HCC patients (Figure 1g and Figure 1h). The above data implied that UBE2T was overexpressed in HCC and was linked to the unfavorable prognosis in HCC patients.

*P < 0.05, ***P < 0.001.

### UBE2T overexpression was markedly linked to the adverse prognosis of HCC patients

**Figure 2.**
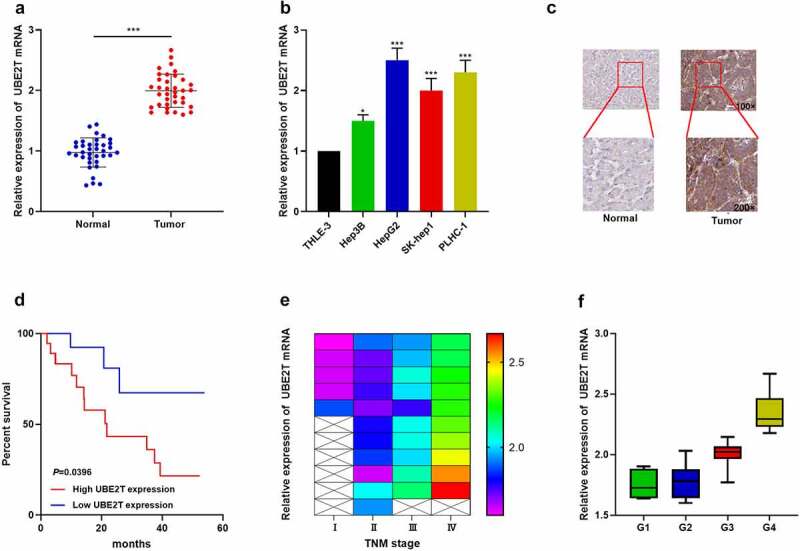
UBE2T overexpression was remarkably linked to adverse prognosis of HCC patients. (a and b) UBE2T expression in HCC tissues and cell lines was detected by qRT-PCR. (c) IHC was executed to detect UBE2T protein expression in HCC tissues and paracancerous tissues. (d) Survival curves were plotted to analyze the relationship between UBE2T expression and overall survival of HCC patients. (e) Correlation between UBE2T expression and TNM stage in HCC patients. (f) Correlation between UBE2T expression and tumor tissue differentiation grade in HCC patients

To further expound the association between UBE2T expression and HCC progression, cancer tissues and paracancerous normal tissues from 36 HCC patients were collected. qRT-PCR indicated that UBE2T expression was remarkably increased in HCC tissues relative to normal tissues (Figure 2a). UBE2T expression was also up-regulated in HCC cell lines (Hep3B, HepG2, SK-help1, and PLHC-1) relative to the normal hepatic epithelial cell line (THLE-3) (Figure 2b). IHC data revealed that, UBE2T protein expression in HCC tissues was also markedly up-regulated compared with paracancerous normal tissues (Figure 2c). Furthermore, UBE2T high expression was positively linked to higher TNM stage and poor differentiation in HCC patients (Figure 2e and Figure 2f, Table 2). Kaplan-Meier curves and multivariate Cox regression analysis unveiled that UBE2T high expression predicted shorter overall survival in HCC patients (Figure 2d, Table 3). The above data indicated that UBE2T was overexpressed in HCC and its overexpression was remarkably linked to adverse prognosis in HCC patients.

**Table 2.** The correlations between clinicopathologic features and UBE2T expression

Pathological indicators	Number of patients	Relative expression of UBE2T		Chi-square value	*P* value
		High expression	Low expression		
All cases	36	18	18		
Age (years)					
<60	18	7	11	1.7778	0.1824
≥60	18	11	7		
Gender					
Male	25	11	14	1.782	0.2777
Female	11	7	4		
Tumor size(d/cm)					
<5 cm	19	10	9	0.1115	0.7385
≥5 cm	17	8	9		
T classification					
T1+ T2	21	7	14	5.6000	0.0180*
T3+ T4	15	11	4		
Lymph node metastasis					
Yes	13	10	3	5.8997	0.0151*
No	23	8	15		
Tumor differentiation					
poorly	20	13	7	4.0500	0.0442*
High	16	5	11

**Table 3.** Variables in the Equation

	B	SE	Wald	df	Sig	Exp(B)	95%CI for Exp(B)
Age	0.355	0.692	0.263	1	0.608	1.426	0.368–5.529
Gender	0.155	0.622	0.062	1	0.803	1.168	0.345–3.953
Lymph node metastasis	−0.950	0.774	1.506	1	0.220	0.387	0.085–1.763
Hepatitis B	−1.201	0.716	2.810	1	0.094	0.301	0.074–1.225
UBE2T expression	1.970	0.776	6.446	1	0.011	7.169	1.567–32.800

*P < 0.05, ***P < 0.001.

### UBE2T enhanced HCC cell proliferation and migration

**Figure 3.**
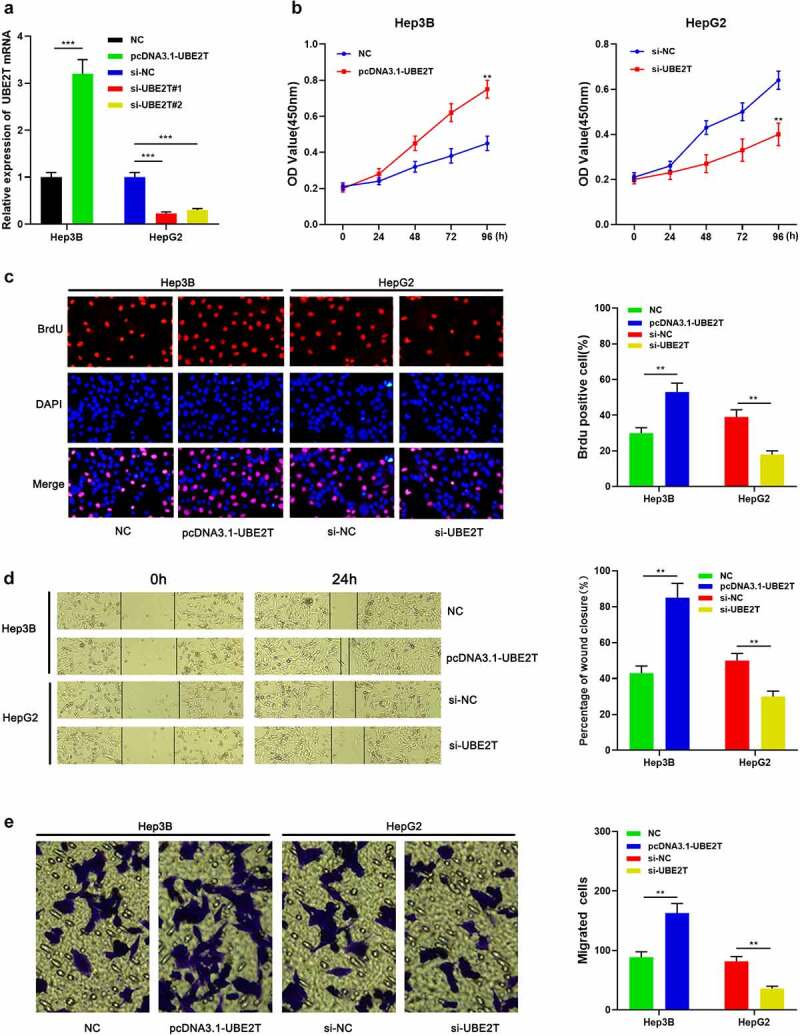
UBE2T enhances the proliferation and migration of HCC cells. (a) pcDNA3.1-UBE2T and si-UBE2T were transfected into Hep3B and HepG2 cell lines, respectively, and the transfection efficiency was detected by qRT-PCR. (b and c) CCK-8 and BrdU experiments are employed to detect the proliferation of HCC cells after transfection. (d and e) Scratch healing and Transwell experiments were executed to detect the migration of HCC cells after transfection

To probe the influence of UBE2T on the malignant phenotypes of HCC cells, Hep3B and HepG2 cells were transfected with pcDNA3.1-UBE2T and si-UBE2T#1/si-UBE2T#2, respectively (Figure 3a). The data of the CCK-8 and BrdU experiments demonstrated that the cell viability of the UBE2T overexpression group were remarkably higher than that of the control group (Figure 3b and Figure 3c). Besides, the data of wound healing assay and Transwell experiment unearthed that the migration of Hep3B cells in UBE2T overexpression group was remarkably higher than that in the control group (Figure 3d and Figure 3e). Conversely, knockdown of UBE2T led to decreased multiplication and migration of HepG2 cells (Figure 3b-Figure 3e). These results revealed that UBE2T worked as an oncogene in HCC.

**P < 0.01, ***P < 0.001.

### UBE2T was the downstream target of miR-212-5p

**Figure 4.**
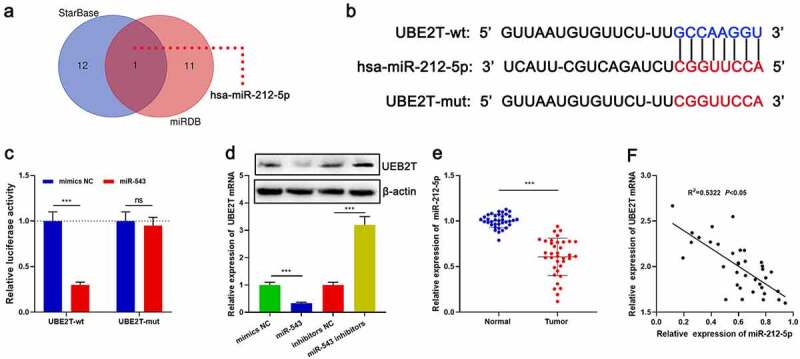
UBE2T was a downstream target of miR-212-5p. (a) StarBase database and miRDB database were applied to predict miRNAs that could interact with UBE2T. (b) The binding site between miR-212-5p and UBE2T 3ʹUTR. (c) Relative luciferase activity was determined 48 h after HEK293T cells were transfected with miR-212-5p mimics/mimics NC and reporter vectors carrying UBE2T sequence. (d) qRT-PCR and Western blot were utilized to detect the effects of miR-212-5p mimics and inhibitors on UBE2T expression in HCC cells. (e) qRT-PCR was performed to detect miR-212-5p expression in HCC tissues. (f) Pearson’s correlation coefficient analysis was used to measure the correlation between miR-212-5p expression and UBE2T expression in HCC tissues

We then tried to determine whether UBE2T could act as a miRNA target. Bioinformatics analysis showed that miR-212-5p was a potential upstream regulator of UBE2T and there was a target site in the UBE2T 3ʹUTR (Figure 4a and Figure 4b). Dual-luciferase reporter gene experiments confirmed a targeted regulatory relationship between miR-212-5p and the UBE2T 3ʹ UTR (Figure 4c). The data of qRT-PCR and Western blot unveiled that miR-212-5p mimics remarkably impeded UBE2T expression relative to the control group; conversely, transfection with miR-212-5p inhibitors remarkably augmented UBE2T expression (Figure 4d). Additionally, miR-212-5p expression was remarkably lower in HCC tissues than in paracancerous tissues, and there was a negative correlation between miR-212-5p expression and UBE2T expression in HCC tissues (Figure 4e and Figure 4f).

***P < 0.001.

ns, no statistical significance.

### miR-212-5p impeded the malignant phenotype of HCC cells via regulating UBE2T

**Figure 5.**
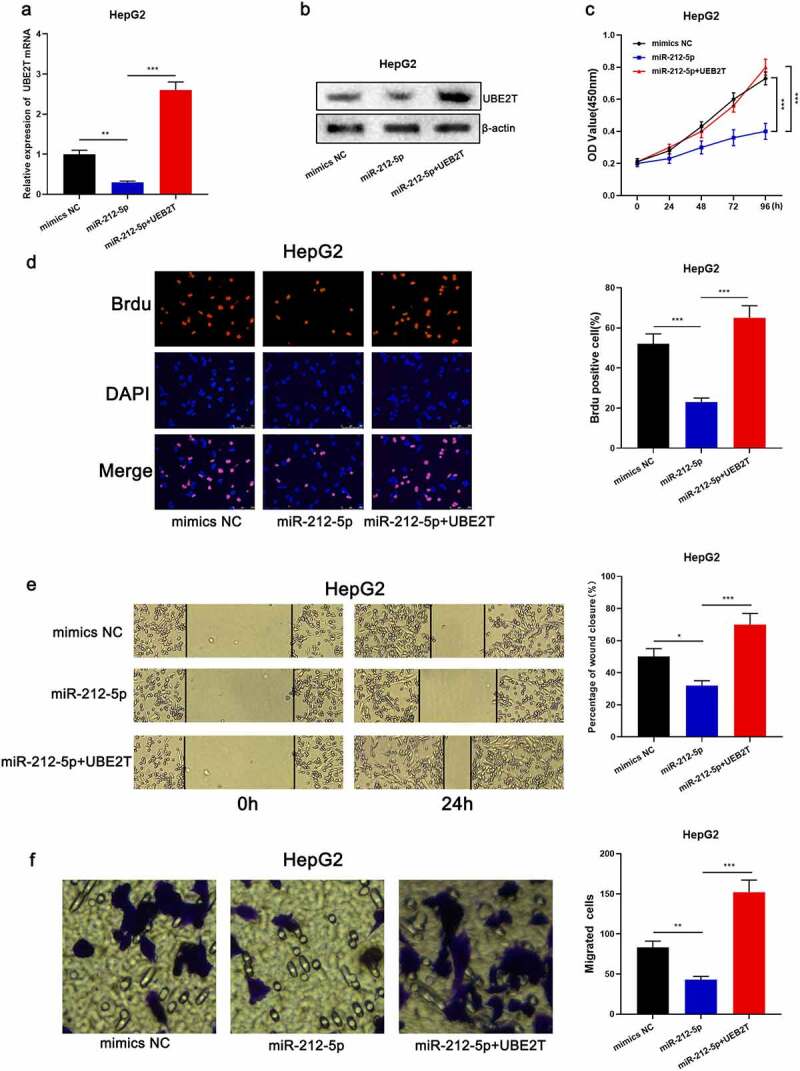
miR-212-5p impeded the malignant phenotype of HCC cells dependent on UBE2T. (a and b) HepG2 cells were transfected with mimics NC, miR-212-5p mimics, and miR-212-5p mimics+pcDNA3.1-UBE2T, respectively, and UBE2T expression in HepG2 cells was detected by qRT-PCR and Western blot. (c and d) CCK-8 and BrdU assay were executed to detect the viability of HCC cells after the transfection. (e and f) Wound-healing assay and Transwell assay were conducted to detect the migration of HCC cells after the transfection

To elaborate on the effects of miR-212-5p and UBE2T on HCC cell proliferation and migration, pcDNA3.1-UBE2T was transfected into HepG2 cells overexpressing miR-212-5p (Figure 5a and Figure 5b). CCK-8, BrdU, scratch healing, and Transwell experiments revealed that the proliferation and migration of miR-212-5p overexpressing cells were markedly decreased relative to the control group, while UBE2T overexpression counteracted the inhibitory effect of miR-212-5p on the multiplication and migration of HepG2 cells (Figure 5c-Figure 5f). The above data implied that miR-212-5p restrained the malignant phenotype of HCC cells in a UBE2T-dependent manner.

*P < 0.05, **P < 0.01, and ***P < 0.001.

## Discussion

The rapid development of molecular biology has provided novel ideas for the diagnosis and treatment of cancer [15]. By working with specific E3 ubiquitin ligase to activate the degradation of related substrates, UBE2T is implicated in multiple biological processes such as cell cycle regulation, signal transduction and tumorigenesis [10]. UBE2T is overexpressed in diverse tumors. For instance, UBE2T facilitates epithelial mesenchymal transition (EMT) via ubiquitination-mediated forkhead box transcription factor O1 (FOXO1) degradation and activating Wnt/β-catenin signaling pathway [16]. UBE2T enhances nasopharyngeal carcinoma cell multiplication and invasion by activating the AKT/GSK3β/β-catenin pathway [17]. Additionally, UBE2T modulates cancer progression by enhancing p53 ubiquitination and degradation in colorectal cancer and HCC [6,10,18]. Wnt/β-catenin signaling pathway is one of the key cascades in regulating physiological and pathological processes, such as cell proliferation and differentiation, stem cell renewal, embryogenesis, and tissue homeostasis [19]. UBE2T is found to potentiate liver cancer stem cells’ functions through direct regulation of Mule-mediated β-catenin degradation [20]. Besides, UBE2T-mediated H2AX/γH2AX monoubiquitination activates CHK1, facilitating DNA repair after radiation, thus contributing to HCC radioresistance [21]. The present study analyzes three HCC-related datasets obtained from the GEO database and unearths that UBE2T expression in HCC tissues is remarkably higher than in paracancerous or normal liver tissues. Subsequently, further analysis is performed with data of TCGA and GEPIA2 databases to verify UBE2T high expression in HCC specimens. UBE2T expression is remarkably higher in the 36 cases of HCC specimens than in the paracancerous tissues, which is consistent with the data of the GEO and TCGA data analysis. Additionally, bioinformatics analysis and experimental data from this work consistently unveil that UBE2T high expression is markedly linked to a worse prognosis in HCC patients. Further in vitro functional experiments reveal that UBE2T enhances HCC cell multiplication and migration. The data suggest the clinical relevance of UBE2T in predicting the prognosis of the patients with HCC, and also imply that targeting UBE2T may block HCC progression.

MiRNAs are short and highly conserved ncRNAs, and they participate in regulating important biological processes. MiRNAs are key regulators of gene expression and can modulate multiple target genes, making them promising candidates for cancer targeted therapy [22,23]. Accumulating research has unearthed that aberrant miRNA expression is strongly related to the development of HCC. For instance, miR-616 is remarkably up-modulated in HCC tissues and cells and it activates the PI3K/AKT pathway through impeding PTEN expression to accelerate HCC progression [9]. MiR-490-3p expression is down-modulated in HCC tissues, and miR-490-3p overexpression restrains HCC cell multiplication and invasion by targeting tropomodulin 3 (TMOD3)[24]. Reportedly, miR-212-5p is abnormally expressed in several human cancers, such as colorectal, lung, stomach, and breast cancers [25–28]. For instance, miR-212-5p restrains the malignant behavior of clear cell renal cell carcinoma cells by targeting transcription factor T-box 15 (TBX15)[29]. MiR-212-5p represses EMT in triple-negative breast cancer by targeting paired related homeobox 2 (PRRX2)[28]. In the present study, bioinformatics and dual-luciferase reporter gene experiments identify and validate UBE2T as a downstream target of miR-212-5p. Furthermore, we demonstrate that, miR-212-5p is remarkably down-modulated in HCC tissues, which is consistent with the previous report [12]. Besides, miR-212-5p expression is negatively correlated with UBE2T expression in HCC tissues. Additionally, we observe that miR-212-5p represses the malignant biological behaviors of HCC cells, and the inhibitory effects of miR-212-5p on the malignancy of HCC cells can be counteracted by UBE2T restoration. These data indicate that miR-212-5p is a tumor suppressor in HCC, and it exerts biological function via repressing UBE2T.

## Conclusion

This work elucidates the link between UBE2T dysregulation and the prognosis and clinicopathological characteristics of HCC patients. Furthermore, UBE2T is demonstrated to facilitate the multiplication and migration of HCC cells and UBE2T can be targeted and modulated by miR-212-5p. Our work is helpful for explaining the mechanism of HCC progression, and offers biomarkers and therapy targets for HCC diagnosis and treatment.

## Supplementary Material

Supplemental Material

## Data Availability

The data used to support the findings of this study are available from the corresponding author upon request.

## Abbreviations

UBE2T, ubiquitin-conjugating enzyme E2T; HCC, hepatocellular carcinoma; TCGA, The Cancer Genome Atlas; GEPIA2, Gene Expression Profiling Interactive Analysis; CCK-8, Cell Counting Kit-8; BrdU, 5-bromo-2ʹ-deoxyuridine; miRNAs, MicroRNAs; mRNA, messenger RNA; PTEN, phosphatase and tensin homolog; PI3K, phosphatidylinositol-3 kinase; TNM, Tumor-Node-Metastasis; DEGs, differentially expressed genes; qRT-PCR, Quantitative Real-time PCR; IHC, Immunohistochemistry; CCK-8, Cell Counting Kit-8; EMT, epithelial mesenchymal transition; FOXO1, Forkhead box transcription factor O1; TMOD3, tropomodulin 3; TBX15, transcription factor T-box 15; PRRX2, paired related homeobox 2.

## Research highlights

1. UBE2T is associated with poor prognosis in hepatocellular carcinoma patients.

2. UBE2T is a target of miR-212-5p.

3. miR-212-5p inhibits hepatocellular carcinoma progression via regulating UBE2T.
