# Supplementary material for: Upregulation of ubiquitin-conjugating enzyme E2T (UBE2T) predicts poor prognosis and promotes hepatocellular carcinoma progression
Source: Bioengineered. 2021 May 3;12(1):1530–42. doi: 10.1080/21655979.2021.1918507 (PMC8806210; doi:10.1080/21655979.2021.1918507)
Supplement: Supplemental Material [file KBIE_A_1918507_SM0310.docx]

| Names | Total | Elements |
| --- | --- | --- |
| StarBase∩miRDB | 1 | hsa-miR-212-5p |
| StarBase | 13 | hsa-miR-498  hsa-miR-196b-5p  hsa-miR-1271-5p  hsa-miR-182-5p  hsa-miR-944  hsa-miR-186-5p  hsa-miR-323a-3p  hsa-miR-299-5p  hsa-miR-577  hsa-miR-96-5p  hsa-miR-196a-5p  hsa-miR-543  hsa-miR-212-5p |
| miRDB | 12 | hsa-miR-4731-3p  hsa-miR-12129  hsa-miR-6806-5p  hsa-let-7c-3p  hsa-miR-891b  hsa-miR-3671  hsa-miR-1277-5p  hsa-miR-5692a  hsa-miR-4801  hsa-miR-5580-3p  hsa-miR-335-3p  hsa-miR-212-5p |

Supplementary Table 1. The candidate miRNA targets predicted by bioinformatics analysis
